# Anatomical and Functional Outcomes of Sutureless Scleral-Fixated Carlevale Intraocular Lens Implantation: A Retrospective Study

**DOI:** 10.3390/jcm14207309

**Published:** 2025-10-16

**Authors:** Adam Słoka, Tomasz Chorągiewicz, Karolina Urbańska, Piotr Więsyk, Marcin Woźniak, Joanna Dolar-Szczasny, Mariusz Spyra, Katarzyna Nowomiejska, Mario Damiano Toro, Robert Rejdak

**Affiliations:** 1 Provincial Eye Hospital in Krakow, 31-723 Krakow, Poland; calicorider@gmail.com; 2 Department of General and Pediatric Ophthalmology, Medical University of Lublin, 20-079 Lublin, Poland; k.urbanska.98@gmail.com (K.U.); marcin.wozniak514@gmail.com (M.W.); joannadolarszczansy@umlub.pl (J.D.-S.); katarzyna.nowomiejska@umlub.pl (K.N.); robert.rejdak@umlub.pl (R.R.); 3 Visum Clinic, Eye Surgery Center in Rzeszów, 35-302 Rzeszow, Poland; marspyra@gmail.com; 4 Public Health Department, Eye Clinic, University of Naples, Federico II, 80138 Naples, Italy; toro.mario@email.it

**Keywords:** cataract surgery, intraocular lens, anterior segment surgery, intraocular lens implantation, intraocular lens surgery, Carlevale IOL

## Abstract

**Background/Objectives**: The purpose of this study was evaluation of the efficacy and the rate of complication of a foldable sutureless scleral-fixated intraocular lens (SSF-IOL), named Carlevale IOL, for the treatment of aphakia without sufficient capsular support due to trauma or complicated cataract surgery. **Methods**: Retrospective, consecutive interventional case series. All consecutive eyes with secondary implantation of aphakic SSF-IOL were considered. The primary outcomes were as follows: best corrected visual acuity (BCVA), refractive error (RE), and intraocular pressure (IOP). Secondary outcome was the occurrence of intraoperative and postoperative complications. **Results**: SSF-IOL was performed in 21 eyes of 21 patients (7 men and 14 women) with mean age of 74 years (range from 36 to 90 years). The mean follow-up time was 11.4 months. VA improved significantly (*p* = 0.0007) from 0.38 logMAR at baseline to 0.11 logMAR at the final follow-up. BCVA improved in 18 patients, remained equal in 2 cases, and worsened in 1 case. Mean postoperative RE was −0.83 Diopters (D) (Median = −0.50 D, SD = 1.05 D) and it was less than 1D in 61.9% of patients. Mean IOP at the end of the follow-up was 15.78 mmHg (ranged from 10 to 22 mmHg, SD = 3.65). In one patient a vitreous hemorrhage was observed intraoperatively, but it resolved spontaneously. Postoperative complications included one case of cystoid macular edema and one case of epiretinal membrane. **Conclusions**: Carlevale SSF-IOL implantation seems to be an effective and safe procedure, ensuring good visual outcomes with a low rate of complication in eyes indicated for secondary IOL implantation.

## 1. Introduction

Despite the progress in development of intraocular surgical techniques of cataract surgery observed in recent decades, aphakia is mostly caused by complications of this procedure. Aphakia commonly results from weakness of zonular fibers, typically due to conditions like pseudoexfoliation syndrome (PEX) or trauma, which increases the risk of intraoperative complications [1,2,3]. Posterior capsule breaks can also manifest at any phase of a scheduled phacoemulsification procedure [4]. In cases without proper capsular support, the implantation of an intraocular lens (IOL) into the ciliary sulcus becomes impracticable.

To date, many techniques for IOL fixation in cases with poor capsular support have been proposed. The IOL may be implanted in the anterior chamber, attached to the iris or sclera, or fixated with a sutureless method [5,6,7,8,9,10,11,12,13,14,15]. Each of these techniques has its own specific advantages, disadvantages, and complications. Thus, in each case the patient’s and surgeon’s individual factors should be considered. The complexity of the surgery and the potential for a poor surgical outcome depend on the positioning of the IOL and the selected fixation technique. For these reasons, nowadays, IOL fixation without capsular support can be challenging.

An ideal method of correction should ensure predictable and stable refractive results in a wide spectrum of aphakic eyes. The operation technique should be relatively easy with a steep learning curve and applicable during primary and secondary operation without any need for extraordinary materials and instrumentation. Moreover, acceptable refraction may be achieved by implanting the IOL in either the anterior or posterior segment of the eye, whether during the primary or secondary operation. The risk of pre- and postoperative complications both in the anterior and posterior segment of the eye should be minimal. The result should be stable in time without risk of dislocation and late complications.

Recently, sutureless IOL scleral fixation has been proposed [16]. The Carlevale lens is a specially designed one-piece acrylic IOL with T-shaped harpoons. These harpoons are resistant to rupture, deformation or displacement following fixation to the sclera. Carlevale IOL implantation is a safe and technically easy procedure which reduces suture-related complications. This lens is foldable so it can be implanted within a narrow corneal tunnel [17].

The aim of this study was to analyze the anatomical and functional outcomes and the rate of intra- and postoperative complications of Carlevale SSF-IOL implantation in posttraumatic and postoperative aphakic eyes.

## 2. Materials and Methods

This is a retrospective interventional surgical case series of consecutive posttraumatic and postoperative aphakic patients at the Department of General and Pediatric Ophthalmology of the Medical University of Lublin from 2017 to 2020. This study followed the tenets of the Declaration of Helsinki. Approval of the Ethics Committee at the Medical University of Lublin, Poland, was given (n° KE-0254/88/2020). All patients were routinely fully informed about the risks and benefits of the surgery, and written consent was obtained. Inclusion criteria were as follows: aphakia after complicated cataract, IOL subluxation or IOL luxation due to trauma. Data was gathered retrospectively from hospital records covering the preoperative, intraoperative, and postoperative periods. Follow-up data for the late postoperative phase were obtained via questionnaires administered at regional ophthalmological offices.

Patients were excluded in cases of active ocular inflammation, infection, or glaucoma. The data collected comprised preoperative and postoperative best corrected visual acuity (BCVA), applanation tonometry measurements, optical coherence tomography (OCT) scans, slit lamp evaluations, and fundus examinations and fundus photography. BCVA was assessed using Snellen charts. Intra- and postoperative complications were gathered to assess the safety of the surgical procedure. Functional success was determined by change in BCVA from baseline to the latest follow-up. The main anatomical outcomes measured included the centration of the optic relative to the pupil and the location of haptic externalization as observed under slit lamp examination.

IOP was measured during every visit using applanation tonometry. All patients underwent standard preoperative biometric measurement by IOL Master 500 (Carl ZeissMeditec, Jena, Germany). The SRK/T formula was used to calculate the power of IOL (A constant = 119.1). For secondary implantations, biometry was performed prior to primary operation. BCVA, IOP, and IOL position were assessed after 4 weeks and 12 months postoperatively, or more often if indicated. Postoperative refraction and keratometry were assessed using the autorefractometer (RK-700 A; Nidek Co. Ltd., Gamagori, Japan). The device has a deviation from the nominal value of ±0.25 D for spherical and cylindrical vertex power ranging from 0.00 to ±10.00 D. The deviation from the nominal value of the ARK Cylinder axis for cylinder power is ±10° for powers between 0.25 D to ±0.50 D, ±5° for powers > 0.5 D to 3.00 D, and ±3° for powers > 3.00 D. Operation duration was measured in minutes.

Postoperatively, steroid (Dexafree^®^, dexamethasone sodium phosphate 1% solution eye drops, Laboratoires Thea, Clermont-Ferrand, France) and antibiotic (Oftaquix^®^, 0.5% levofloxacin ophthalmic solution, Santen Oy, Tampere, Finland) were given five times per day for 2 weeks and then only steroid three times per day for the following 2 weeks.

### 2.1. Surgical Technique

All procedures were performed under local anesthesia. In cases involving a luxated lens or intraocular lens, a standard 25-gauge pars plana vitrectomy was conducted (4 patients). Intraoperative intraocular pressure was maintained using an anterior chamber maintainer inserted through a corneal paracentesis.

A 3 mm conjunctival peritomy was created at both the nasal and temporal meridians. Following scleral exposure at the 0° and 180° meridians, a 1 mm-deep intrascleral pocket was created using a crescent blade, beginning 3 mm posterior to the limbus. Within this pocket, a perpendicular sclerotomy was created 2 mm posterior to the limbus using a 25-gauge needle.

A 2.2 mm clear corneal tunnel incision was made at the 12 o’clock position. The Carlevale IOL was then carefully injected into the anterior chamber using a Medical Viscojet 2.2 mm injector. As only the leading haptic emerged from the injector tip, the corresponding plug was grasped with 25-gauge vitrectomy forceps (Alcon) inserted through the previously prepared sclerotomy. The IOL was simultaneously advanced from the injector and gently pulled with the forceps, ensuring a controlled and coordinated delivery of the haptic into the scleral pocket.

**Figure 1:**
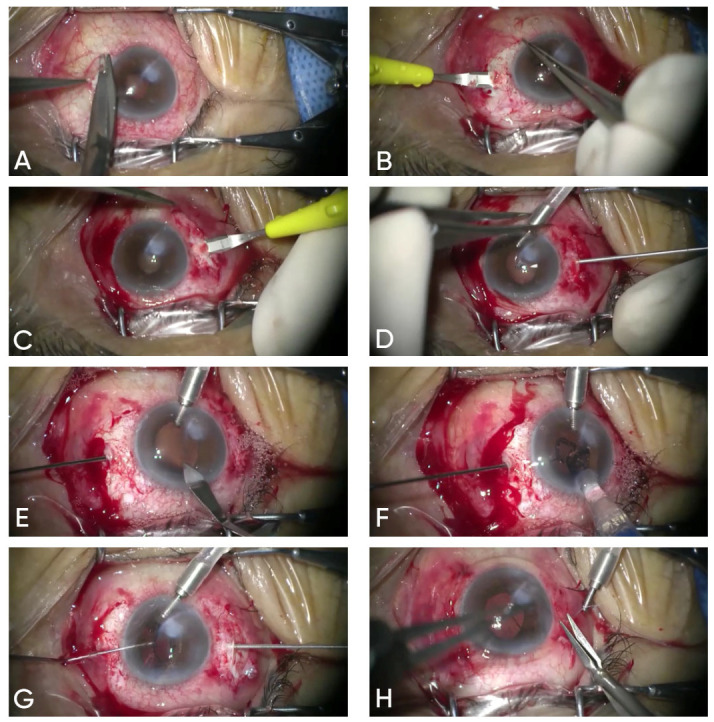
The graphics show the surgical technique of sutureless scleral-fixated Carlevale intra-ocular lens implantation: (**A**) A 3 mm conjunctival peritomy at 3 and 9 o’clock positions. (**B**) Creation of 1 mm intrascleral pocket at 0°, 3 mm posterior to the limbus. (**C**) Intrascleral pocket at 180°, 3 mm from limbus. (**D**) Perpendicular sclerotomy (25 G needle), 2 mm posterior to the limbus. (**E**) A 2.2 mm clear corneal incision at 12 o’clock; insertion of 25 G vitrectomy forceps. (**F**) IOL injected; leading haptic grasped and externalized. (**G**) Trailing haptic grasped and externalized; both fixed in scleral pockets. (**H**) Conjunctival closure with single 8-0 Vicryl suture.

Once the IOL was fully positioned within the anterior chamber, the trailing plug was similarly grasped and externalized through the contralateral sclerotomy. The corneal tunnel incision was then hydrated, and the scleral pockets were covered with conjunctiva, which was secured using an 8-0 Vicryl suture (Figure 1).

### 2.2. Statistical Analysis

Statistical analysis was performed using R programming language (version 4.3.2) and RStudio IDE for R language (version 2023.09.1). All statistical tests were performed with 95% statistical significance. Statistically significant differences were defined as those with *p*-values less than 0.05. The Shapiro–Wilk test was used to assess the normality of distributions, while the Wilcoxon test was employed to evaluate the improvement of VA. Student’s *t*-test was used to examine change between preoperative IOP and first postoperative IOP. Student’s *t*-test was used to examine change in preoperative IOP and last follow up IOP.

## 3. Results

The study comprised 21 eyes of 21 patients (14 females and 7 males). The mean age of the patients was 74 years (range 36 to 90 years; median 74.00 years, SD = 10.90 years). The mean follow-up time was 11.4 months (ranging from 2.7 to 20 months; median 11.43 months, SD = 6.25 months). Indications for secondary IOL implantations were the following: aphakia after complicated cataract surgery (12 patients) and IOL’s subluxation/luxation (9 patients). Summary of the results can be found in Table 1.

### 3.1. Visual Acuity

BCVA improved in 18 cases, remained the same in 2 cases, and worsened in 1 case. Mean VA improved significantly (*p* = 0.0007) from 0.38 Logarithm of the Minimum Angle of Resolution (logMAR) (median = 0.4 logMAR, SD = 0.33 logMAR) at baseline to 0.11 logMAR (median 0.05 logMAR, SD = 0.11 logMAR) at the final follow-up.

### 3.2. Refraction

The mean postoperative refractive error (RE) characterized by the variance between target refraction and spherical equivalent of postoperative refraction was −0.83 D (range from −3.75 D to 0.75 D; median = −0.5 D, SD = 1.05 D). It was within the range of 1 D in 61.9% of eyes. The achieved refraction data are distributed normally (*p* = 0.18).

### 3.3. Intra- and Postoperative Abnormalities and Complications

There was one intraoperative complication—moderate vitreous hemorrhage in one patient, which resolved spontaneously. The rest of surgeries were completed uneventfully in both groups. Postoperatively, no decentration was observed. During postoperative follow-up, abnormalities were observed in two eyes (9.5%). CME was observed in one eye (4.8%). Another complication was epiretinal membrane (ERM) which occurred in a patient with IOL luxation after YAG capsulotomy. ERM was diagnosed during the postoperative follow-up period. The mean duration of operation was 48.80 min (range from 29 to 96 min, median = 40.00 min, SD = 20.91 min)

### 3.4. IOL Position

All patients were clinically evaluated at each follow-up, and no cases of decentration, significant tilt, luxation or harpoon exposure were observed.

### 3.5. Intraocular Pressure

The mean IOP at the end of the observation period (15.78 mmHg median = 16.40 mmHg, SD = 3.65 mmHg) was not significantly changed (*p* = 0.40) in comparison to the mean preoperative IOP (16.81 mmHg median = 16.00 mmHg, SD = 4.17 mmHg). There was no eye with postoperative IOP higher than 22 mmHg. Mean IOP at the first postoperative visit was 16.62 mmHg (median = 17.00 mmHg, SD = 3.09 mmHg), which was not significantly lower than preoperative IOP (*p* = 0.87). The lowest value was 10 mmHg. In no cases was a positive Seidel test observed. There were no signs of wound leakage, hypotony, or need for wound suturing.

## 4. Discussion

In this study, the anatomical and functional results after implantation of a scleral-fixated, foldable, sutureless Carlevale IOL are described. This recently introduced technique offers numerous advantages compared to conventional transscleral suturing of the IOL. The Carlevale lens is the first lens designed and adapted to functional, anatomical, and surgical requirements.

A worldwide growing branch of ophthalmology is linked with achieving acceptable refraction in eyes with poor or without capsular support. These conditions may manifest after the lens or IOL luxation resulting from trauma or insufficient zonular fibers. Previous studies indicate that different surgical procedures have been shown to be effective; however, the postoperative instability of the IOL which could cause late complications is still a problem [18,19,20]. Therefore, new projects of IOLs and their implantation techniques are under development. A notable modification of Carlevale IOL fixation is the technique described by Manousakis et al. [21], which eliminates conjunctival manipulation by employing Hoffman pockets rather than conjunctival openings, thereby potentially reducing the risk of postoperative infection and promoting faster recovery [21]. Another recently described technique involves creating partial-thickness scleral grooves, 2 mm from the limbus, to guide and secure the T-shaped haptic anchors intrasclerally. This method has demonstrated safety, efficacy, and stable centration in a series of 47 aphakic eyes [22].

Performing intrascleral sutureless IOL fixation is a relatively intricate procedure that could pose challenges and require additional time for beginners or surgeons with limited experience. A steep learning curve might be required. This lens, as a hydrophilic acrylic IOL, theoretically has good uveal biocompatibility and great foldability. The specially designed T-shaped harpoons, when fixed to the sclera, demonstrate greater resistance to rupture, deformation, or dislocation compared to the haptic of a 3-piece IOL [17]. The most important phase of this surgery is the externalization of T-shaped harpoons which minimize IOL decentration and torsion combined with the implantation of the IOL into the anterior chamber. On the other hand, the relatively high cost of the IOL reduces the popularity of this technique. Symmetric positioning of the sclerectomies for harpoons and unique haptics specially designd for scleral fixation in wide range of eye dimensions provide a stable and predictible position of the IOL. Although quantitative measurements were not collected, no cases of IOL decentration were observed. Hydrophilic acrylic material could be potentially fragile during manipulation, especially while pulling through the sclera. In the presented case series, no case of haptic destruction was observed. The size of scleral pockets ensured proper seal covering of harpoons. During the time of observation, no cases of harpoon exposure, hypotony or tissue inflamation were observed.

The IOL fixation technique invented by Yamane is described as a new mini-invasive technique of sutureless intrascleral fixation [15]. To externalize the haptics of a three-piece IOL, two needles are employed. A flanged haptic tip is created and subsequently cauterized to facilitate intrascleral fixation. Risk of post-operative hypotony using this technique is decreased due to minimizing diameter of the sclerotomies and its tunnel-like, self-sealing architecture [23]. The Yamane technique has already been reported in postoperative aphakia due to complicated cataract surgery, trauma or dislocated IOL [23,24]. On the other hand, this technique has a relatively steep learning curve; a symmetrical parallel limbus scleral tunnel is essential for achieving the correct positioning of the 3-piece IOL. Manipulating the haptics is almost invariably required to adjust IOL centration and may lead to complications such as haptic damage, which may compromise the success of the surgery [25].

Another technique for sutureless scleral fixation is the one introduced by Canabrava et al. [26] known as the four-flanged technique using a four-eyelet foldable IOL. In this method, two transconjunctival sclerotomies are made 180 degrees apart, approximately 2 mm posterior to the limbus, using a 30-gauge needle. A 5-0 polypropylene suture is threaded through the eyelets and the suture ends are externalized through the sclerotomies. Flanges are created using thermocautery and inserted inside the scleral tunnels [26,27]. Four-flanged scleral IOL fixation provides consistent and effective results [27,28]. Moreover, it overcomes the challenges of haptic engagement and externalization seen in the Yamane technique, without requiring a specialized 30-gauge thin-walled needle [29]. However, further studies on the outcomes and complications of the technique are needed to fully establish its safety.

Giannopoulos et al. [22] recently described a novel technique involving the creation of two partial-thickness linear scleral grooves positioned 180 degrees apart and located 2 mm posterior and parallel to the limbus. In the middle of each groove, a full-thickness sclerotomy is performed to facilitate the externalization of the Carlevale intraocular lens anchors. These grooves enable secure lodging of the anchors within the sclera by allowing them to plug the full thickness sclerotomies without protrusion. This modified approach to sutureless scleral fixation demonstrates both safety and efficacy. The authors suggest that the technique is less time-consuming and technically more straightforward than previous methods, potentially representing a valuable option for future surgical practice [22].

The technique developed by Mr. Sushrutha Dissanayake and described by Stewart et al. [30] introduces several key modifications to the original Carlevale technique. Instead of scleral flaps, the method involves creating linear partial-thickness scleral incisions measuring 3 to 4 mm in length and positioned 1.5 mm posterior to the limbus using a No. 11 scalpel blade. A 23-gauge MVR knife is used to create a sclerotomy to access the vitreous cavity. The Carlevale lens is then injected into the anterior chamber using an Acujet injector via a 2.75 mm keratome incision. The leading haptic is grasped and externalized using Carlevale forceps, and the trailing haptic is delivered using a handshake technique involving Eckardt’s forceps. Notably, the Eckardt forceps are only used to transfer the haptic to the Carlevale forceps, avoiding direct externalization to prevent potential haptic damage. Once the haptics are positioned in the scleral grooves, the scleral incisions are sutured with 8–0 Vicryl sutures. The conjunctiva is also closed with the same suture. This adaptation maintains the stability of the IOL without relying solely on the self-locking mechanism of the haptics, potentially lowering the risk of extrusion. Additionally, suturing the scleral incision provides a watertight seal, reducing the risk of postoperative hypotony. In the clinical experience described, this modified approach resulted in favorable refractive outcomes and no haptic extrusion over a follow-up period exceeding one year. Overall, while the original Carlevale technique offers a sutureless and efficient approach with proven success, Dissanayake’s modified method provides a simplified and secure alternative, particularly in cases where flap creation may be technically challenging or when additional stability is desired through scleral suturing [30].

The modified, less invasive technique described by Danese et al. [31] involved posterior vitrectomy combined with Carlevale intraocular lens (IOL) implantation. The procedure was performed using a modified approach for cannula placement: one 25-gauge cannula was inserted into the pars plana for posterior segment access, while two additional cannulas were placed into the ciliary sulcus to facilitate anterior support and manipulation. Following completion of the pars plana vitrectomy, the Carlevale IOL was implanted via a standard injector system. Both T-shaped haptic anchors were carefully externalized through scleral incisions and positioned beneath the conjunctiva, ensuring stable IOL fixation without the need for sutures. Crucially, this method avoids large scleral dissections and sutures, reducing surgical invasiveness [31]. The study—evaluating 35 eyes over ~2 years—demonstrated good lens centration, stable anatomical outcomes, and no conjunctival erosion [32].

In this study, 85.71% of patients noted an improvement in visual outcomes. Mean BCVA was 0.38 logMAR at baseline and significantly improved to 0.11 logMAR at the final follow-up. Our outcomes stand with other studies using the Carlevale lens where BCVA ranged from 0.09 logMAR to 0.44 logMAR in the last follow-up [16,28,33,34,35,36,37,38,39,40,41]. Three eyes did not show visual improvement. One eye had a preoperative BCVA of 1.0 (decimal), which remained stable postoperatively. Another eye showed no change in BCVA due to pre-existing advanced age-related macular degeneration (AMD), while the third eye worsened, also in the context of AMD and progressive retinal pathology unrelated to the surgical procedure. These two cases highlight the influence of underlying retinal disease on functional outcomes, despite successful anatomical positioning of the IOL.

The mean postoperative RE in our study was −0.83 D. RE in the other studies ranged from −0.24 D to −0.5 D [33,37,38,41]. The summary of visual and refractive outcomes in the other studies using the Carlevale lens can be found in Table 2. However, RE can be influenced by several risk factors such as the presence of ocular comorbidity, low preoperative VA, and previous ocular surgery, which may worsen refractive outcomes [42]. Therefore, the reliance on RE to evaluate the effectiveness of the technique should be avoided. Implantation of Carlevale SSF-IOLs has comparable outcomes to those achieved with other secondary IOL implants currently available, such as anterior chamber IOLs, iris fixation IOLs (anterior/posterior/suture), and scleral fixation IOLs (suture/sutureless) [43]. Seknazi et al. reported less postoperative induced astigmatism and less RE of the Carlevale lens in comparison to iris claw lens [40].

Some studies have shown that implantation of the Carlevale lens is safe and fast [16]. However, in our study the mean operation duration was approximately 50 min, which may be attributed to the inclusion of four cases that involved PPV. After excluding these cases, the mean surgical time was 41 min, ranging from 29 to 65 min, which more accurately represents the expected operative time for isolated Carlevale lens implantation. Importantly, we observed that all PPV cases showed improvement in BCVA, indicating positive functional outcomes despite the added surgical complexity. For this reason, these cases were not excluded from the BCVA analysis, as their inclusion reflects the real-world effectiveness of the procedure, even in more complex surgical scenarios.

The lack of implant suturing and haptic manipulation preserves the conjunctiva and reduces the risk of suture-related and haptic damage complications (e.g., iris or cornea lesion, irritation of uvea—uveitis–glaucoma–hyphema syndrome) [44,45]. All techniques involving sclera incisions create a risk of vitreoretinal tractions and secondary retinal detachment as a final effect. Carlevale IOL implantation is based on the use of blunt ophthalmic instruments which decreases the risk of some intraoperative complications. Due to the self-blocking mechanism and the harpoon-like plugs, which are secured and anchored within the scleral bed underneath the sutured scleral flaps, these lenses demonstrate exceptional stability [45]. Hypotony, secondary to leakage from the sclerotomies, is one of the most frequent postoperative complications [35]. However, we have not observed any cases of hypotony during the follow-ups [35]. Furthermore, we have not noted any cases of IOL dislocation during the follow-up. CME represents one of the most common postoperative complications [46]. One case of postoperative CME was reported in our study (4.8%). In other studies, using the Carlevale lens, the incidence of CME ranged from 3.1% to 7.4% [16,34,38]. Macular edema following retropupillary IOL fixation is noted in 1.2% to 8.7% of cases [47,48]. The same frequency occurs in the case of anterior chamber iris–claw IOL [49]. The occurrence of macular edema in our study is comparable to that observed with transscleral fixated posterior chamber intraocular lens (PCIOL), where the rate of edema was 10.4% [50]. In cases of anterior chamber intraocular lens (ACIOL), macular edema was reported in 2.7% patients [51]. Carlevale SSF IOL delivers positive functional outcomes with a minimal incidence of postoperative complications [43]. Excellent outcomes in terms of intraocular lens (IOL) centration and stability have been demonstrated with the Carlevale fixation technique for scleral-sutureless (SSF) IOLs [52]. In a prospective study with a mean follow-up of approximately 28 months, Schranz et al. [53] reported sustained IOL stability, with a slight decrease in decentration (from 0.41 mm to 0.39 mm) and a modest increase in lens tilt (by ~0.5°, to ~7.7°). Both changes were statistically significant [53].

Long-term imaging data suggest that scleral pockets covering the haptics may undergo structural changes over time. In the study by Schranz et al. [53], anterior segment optical coherence tomography (AS-OCT) revealed significant thinning of the scleral pocket overlying each T-haptic over a period of 2–3 years, at an approximate rate of 0.03 mm per year. By around three years postoperatively, the pockets were measurably thinner. The authors concluded that this finding raises concerns about the long-term integrity or “patency” of these scleral pockets [53].

Reported complications associated with Carlevale IOL fixation are relatively few. Haptic extrusion and conjunctival erosion are rare but documented events. In long-term studies involving scleral pockets or flaps, isolated cases of haptic anchor extrusion have been reported. For example, in one comparative study, 3 of 59 eyes with scleral pockets exhibited haptic plugs located outside their intended pockets, beneath the conjunctiva [54]. Similarly, Fiore et al. [55] observed plugs positioned outside the pockets in 13% of eyes, including one instance of plug tip fracture; however, no cases of IOL decentration were noted [55]. However, further studies are necessary to evaluate the long-term stability and durability of SSF IOL fixation.

## 5. Conclusions

In conclusion, this surgical technique of Carlevale SSF IOL is safe and effective in providing good visual and refractive outcomes in patients who require secondary IOL implantation. The main limitation of our study is its retrospective design and the absence of regular OCT screening. Moreover, the relatively small sample size, limited follow-up duration, and lack of quantitative data on lens centration and tilt further constrain the robustness of our findings. A longer follow-up period and a larger surgical series are necessary to comprehensively validate the safety, advantages, and limitations of this novel surgical technique.

## Figures and Tables

**Table 1:** Summary of the results.

Result	Mean	Median	Standard Deviation
Preoperative visual acuity	0.38 logMAR	0.40 logMAR	0.33 logMAR
Postoperative visual acuity	0.11 logMAR	0.05 logMAR	0.11 logMAR
Refractive error	−0.83 D	−0.5 D	1.05 D
Preoperative intraocular pressure	16.81 mmHg	16.00 mmHg	4.17 mmHg
Postoperative intraocular pressure	15.78 mmHg	16.40 mmHg	3.65 mmHg

**Table 2:** Summary of visual and refractive outcomes in the other studies using the Carlevale lens.

Authors	Sample Size (Eyes)	BCVA Preoperatively [logMAR]	BCVA at the Last Follow-Up [logMAR]	Mean RE
Sidiropoulos et al. [33]	27	0.85 ± 0.59	0.44 ± 0.30 at 1 month postoperatively 0.36 ± 0.34 at 6 months postoperatively	−0.5 ± 0.99 D
Vaiano et al. [34]	54	0.93 ± 0.61	0.42 ± 0.34 at 3 months postoperatively 0.42 ± 0.37 at 6 months postoperatively 0.38 ± 0.38 at 12 months postoperatively	
Rouhette et al. [35]	72	0.52 ± 0.5	0.15 ± 0.6	
Georgalas et al. [36]	169	0.58 ± 0.49	0.09 ± 0.1	
Fiore et al. [37]	18		0.41 ± 0.33	−0.31 ± 0.71 D
Barca et al. [38]	32	0.46 ± 0.29	0.13 ± 0.12	−0.24 ± 0.81 D
Gabai et al. [39]	13	0.75 ± 0.5	0.28 ± 0.3	
Rossi et al. [16]	78	0.86 ± 0.56	0.38 ± 0.42 at 6 months postoperatively	
Seknazi et al. [40]	44		0.14 ± 0.11	
Franco et al. [41]	28	0.78 ± 0.37	0.23	−0.33 D
Schranz [28]	29		0.13 ± 0.57	

## Data Availability

The original contributions presented in this study are included in the article. Further inquiries can be directed to the corresponding author.

## Abbreviations

SSF-IOL	Sutureless scleral-fixated intraocular lens
VA	Visual acuity
RE	Refractive error
IOP	Intraocular pressure
logMAR	Logarithm of the Minimum Angle of Resolution
D	Diopter
CME	Cystoid macular edema
ERM	Epiretinal membrane
PEX	Pseudoexfoliation syndrome
BCVA	Best corrected visual acuity
OCT	Optical coherence tomography
PCIOL	Posterior chamber intraocular lens
ACIOL	Anterior chamber intraocular lens
